# Benznidazole Treatment of Chagasic Encephalitis in Pregnant Woman with AIDS

**DOI:** 10.3201/eid1909.130667

**Published:** 2013-09

**Authors:** Margarita Bisio, Jaime Altcheh, Jorge Lattner, Guillermo Moscatelli, Valeria Fink, Juan M. Burgos, Facundo García Bournissen, Alejandro G. Schijman, Héctor Freilij

**Affiliations:** Hospital de Niños Ricardo Gutiérrez, Buenos Aires, Argentina (M. Bisio, J. Altcheh, G. Moscatelli, F. García Bournissen, H. Freilij);; Instituto de Investigaciones en Ingeniería Genética y Biología Molecular Dr Héctor N. Torres, Buenos Aires (M. Bisio, A.G. Schijman);; Hospital Juan A. Fernández, Buenos Aires (J. Lattner, V. Fink);; Facultad de Medicina de la Universidad de Buenos Aires, Buenos Aires (J.M. Burgos)

**Keywords:** Trypanosoma cruzi, HIV, Chagas disease reactivation, pregnancy, benznidazole, parasites, protozoa, AIDS

## Abstract

We report a case of chagasic meningoencephalitis reactivation in a pregnant woman co-infected with *Trypanosoma cruzi* and HIV that was successfully managed with benznidazole and highly active antiretroviral therapy. Early diagnosis enabled rapid specific treatment that improved the health of the patient and her baby.

Chagas disease, caused by the protozoan *Trypanosoma cruzi*, is transmitted to humans mainly by triatomine bugs (vector-borne route), through blood transfusions, or from mother to child through the placenta (transplacental route) (*1*). In patients co-infected with HIV, Chagas disease reactivation generally occurs in persons with CD4 T-cell counts <200 cells/mm^3^ and results in severe meningoencephalitis or myocarditis (*2*). Confirmation of central nervous system (CNS) reactivation requires *T. cruzi* detection in 1) cerebrospinal fluid, 2 brain tissue, or 3) blood, with neurologic manifestations and clinical response to parasiticidal treatment (*2*). CNS reactivation is associated with a high mortality rate, and management consists of combining anti–*T. cruzi* drugs (benznidazole or nifurtimox) with highly active antiretroviral therapy (HAART) to favor immune reconstitution (*2*,*3*).

Migration by persons from Chagas disease–endemic areas to vector-free urban centers, and changes in the epidemiologic profile of HIV, have led to a large overlap in the geographic distribution of the 2 infections (*4*). In fact, the prevalence of *T. cruzi* infection among HIV-seropositive patients from disease-endemic regions was found to be 1.3% in Brazil and 1.9% in Spain (*4*,*5*).

Because safety of benznidazole in pregnancy has not been established (*6*), its use in treating pregnant women is contraindicated (*7*,*8*). Prevalence of vertical transmission of *T. cruzi* infection from immunocompetent women to their fetus varies from 0.1% to 18% among regions (*7*), and such transmission is strongly associated with the maternal blood-parasite load (*7*,*9*). However, patients co-infected with HIV exhibit higher levels of parasitemia and a higher congenital transmission rate (*10*) than those who are not co-infected. Indeed, in our experience, 6 of 7 co-infected pregnant women transmitted *T. cruzi* infection (*11*). We describe a noteworthy case-patient from that series, a woman who experienced reactivation of *T. cruzi* infection during the third trimester of pregnancy but did not transmit the parasite infection, probably because she received, without delay, treatment with benznidazole and HAART.

## The Patient

A 33-year-old woman, who had been infected with HIV for 11 years, started receiving lamivudine, zidovudine, and nevirapine at week 26 of pregnancy. At that time, CD4 cell count was 18 cells/mL. She had begun receiving HAART 1 year before pregnancy but abandoned treatment after a few months. Her compliance with treatment, laboratory tests, and follow-up was poor.

At week 32 of pregnancy, she was admitted to the hospital with clinical manifestations of intracranial hypertension and meningoencephalitis (temporospatial disorientation, slurred speech, nausea and vomiting, and hemiparesis). A CNS mass was detected by magnetic resonance imaging (Figure). Results of serologic tests were negative for *Toxoplasma gondii*, *Treponema pallidum*, and hepatitis B and C viruses but positive for *T. cruzi*. Reactivation of Chagas disease was confirmed by a positive microhematocrit measurement (*12*). PCR targeted to *T. cruzi* kinetoplast DNA was positive, and quantitative PCR (qPCR) targeted to satellite *T. cruzi* DNA yielded 308.9 parasite equivalents/mL of blood (*13*). The parasite genotype (discrete typing unit) detected by PCR strategies was identified as TcV (*14*). Most probably, this patient acquired the infection by the vector-borne route in a highly disease-endemic province in Argentina where she had lived as a child.

**Figure:**
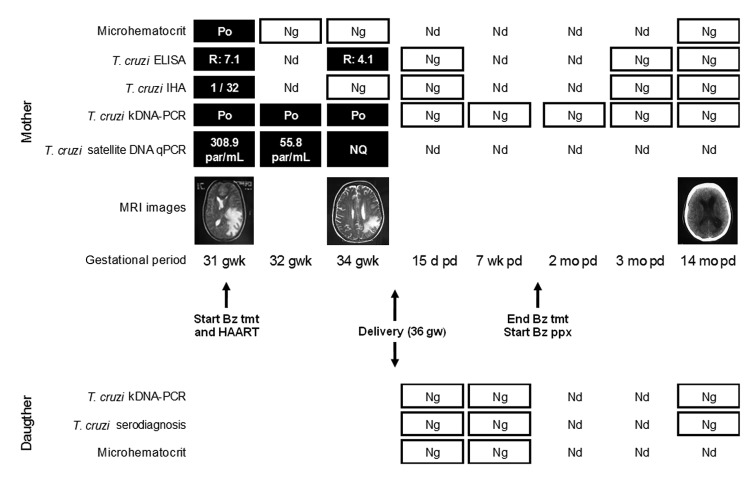
Diagnosis, treatment, and prophylaxis monitoring of Trypanosoma cruzi infection in a pregnant woman and her daughter. The mother, who had chagasic encephalitis and AIDS, was treated with benznidazole (Bz). Results of ELISA were considered positive when R was >1.2); results of IHA were considered positive when titers were >16. Po, positive; Ng, negative; Nd, not determined; IHA, indirect hemagglutination assay; kDNA-PCR, kinetoplast DNA-PCR; qPCR, quantitative PCR (dynamic range of detection: 1–105 parasite equivalents/mL, amplification efficiency 0.95, R2 = 0.996); par/mL, parasite equivalents/mL of blood; NQ, positive but not quantifiable; MRI, magnetic resonance imaging; gwk, gestational week; pd, postdelivery; tmt, Bz treatment (5 mg/kg/d); HAART, highly active antiretroviral therapy; ppx, Bz prophylaxis (5 mg/kg/3× per week).

Although data on benznidazole use in pregnancy are lacking, after an in-depth discussion of potential risks and benefits of the treatment, we concluded that, because of her severe clinical condition, treatment with benznidazole could be beneficial. Within 48 hours of the diagnosis, she received benznidazole (5 mg/kg/day, for 84 days) without side effects. The patient showed rapid neurologic and clinical improvement. Parasitic loads, determined by qPCR, dropped rapidly (Figure). During treatment, results of the microhematocrit, PCR, and anti–*T. cruzi* serologic testing became negative for the parasite. Magnetic resonance images showed substantial reduction of edema and the mass and reconstitution of the interventricular line without evidence of ventricular dilatation (Figure). Seven weeks after treatment initiation, cerebrospinal fluid was negative for *T. cruzi* by serologic testing, microhematocrit, and PCR.

At week 36 of gestation, the patient gave birth to a girl by elective cesarean section. The baby’s birthweight was low for gestational age (1,700 g, <3rd percentile), and her Apgar score was 7/8. The newborn was hospitalized for 32 days. Microhematocrit and PCR results suggested that she had not acquired *T. cruzi* infection. This lack of infection was confirmed by means of serologic testing at 1 year of age, per current Chagas disease guidelines (*12*). Moreover, results of PCR for HIV and p24 antigen tests were negative in 2 different samples, enabling perinatal infection with HIV to be ruled out.

After delivery, the mother continued to receive HAART with benznidazole prophylaxis (5 mg/kg/day 3×/week) for 6 months. A follow-up computed tomographic scan performed 14 months after delivery showed a favorable response. Moreover, serologic, parasitologic, and molecular studies remained negative for *T. cruzi* (Figure). To date, the patient continues to show erratic compliance with follow-up. As a consequence, immunologic recovery has not yet been achieved.

## Conclusions 

Benznidazole is traditionally considered contraindicated in pregnancy because data that support its safety to the fetus are lacking (*8*). Nevertheless, because of the severe clinical picture of the patient, and the known lower risks of fetal toxicity in the second and third trimesters of pregnancy (*6*), treatment was administered on a compassionate basis. Among patients who have AIDS and Chagas disease reactivation with CNS involvement, early parasiticidal and HAART treatment can improve the poor prognosis (*2*,*3*). Cordova et al. (*2*) reported a mortality rate of 79% in a cohort of 15 patients with co-infection, with a median time of survival of 21 days after hospitalization. However, the researchers associated this high mortality rate with delayed diagnosis, because it took a median of 18 days to recognize the condition. In the patient reported here, the short interval (72 hours) between admission and initiation of benznidazole treatment may have improved the chances of a successful outcome. Moreover, to our knowledge, the patient in this case has longest reported survival time after diagnosis of chagasic reactivation caused by HIV co-infection, with 7 years of follow-up (*15*).

Microhematocrit and PCR successfully demonstrated the presence and elimination of *T. cruzi,* and qPCR enabled the decrease in parasitic load to be monitored. *T. cruzi* TcV detected in peripheral blood was described as prevalent in the population from the Southern Cone (*14*). Notably, negative serologic results were achieved early after treatment, which is only usually seen in immunocompetent patients at the acute phase of disease or in recent congenital infections. The rapid negative seroconversion observed in this patient is uncommon. The continued seronegative results when her immune system presumably was reconstituted suggests strongly that she had an actual parasitologic cure.

Moreover, the baby’s weight at birth was low for gestational age. Various reasons could explain the low birthweight, including the infectious status of the mother or the severe stress she was experiencing at the time of admission. Also, a potential effect of benznidazole for the baby cannot be fully ruled out. Nonetheless, the benefit to both mother and child clearly outweighed the risk. The baby did not acquire either infection. 

This case illustrates that parasiticidal treatment may decrease parasitic load and prevent vertical transmission of *T. cruzi*, even in co-infected patients among whom a higher incidence of congenital infection and illness of newborns is observed (*10*). This result reinforces the idea that benznidazole has a role in the prevention of congenital transmission of Chagas disease. Therefore, studies that explore its safety and effectiveness during pregnancy may be warranted.
